# Spontaneous preterm labor is associated with an increase in the proinflammatory signal transducer TLR4 receptor on maternal blood monocytes

**DOI:** 10.1186/1471-2393-10-66

**Published:** 2010-10-21

**Authors:** Edyta Pawelczyk, Bogdan J Nowicki, Michael G Izban, Siddharth Pratap, Nupur A Sashti, Maureen Sanderson, Stella Nowicki

**Affiliations:** 1 The National Institutes of Health, Bethesda, Maryland, USA; 2 The Departments of Obstetrics & Gynecology, Meharry Medical College, Nashville, TN, USA; 3 Department of Microbiology and Immunology, Meharry Medical College, Nashville, TN,USA; 4 Communicable and Environmental Disease Services, Tennessee Department of Health, Nashville, TN, USA

## Abstract

**Background:**

Localized inflammation and increased expression of TLR4 receptors within the uterus has been implicated in the pathogenesis of preterm labor. It remains unclear whether intrauterine inflammatory responses activate the maternal peripheral circulatory system. Therefore we determined whether increased TLR4 expression is present in the peripheral maternal white blood cells of women with spontaneous preterm labor.

**Methods:**

This is a cross-sectional study of 41 preterm labor cases and 41 non-preterm controls. For each case and control sample, RNA was purified from white blood cells and TLR4 mRNA pool size was evaluated by quantitative PCR. Protein expression levels were determined by flow cytometry. Statistical evaluation using multiple linear regressions was used to determine any significant differences between the cases and controls. The purpose was to determine association prevalence of TLR4 levels and preterm labor.

**Results:**

Adjusted mean TLR4 mRNA levels of 0.788 ± 0.037 (standard error) for preterm labor and 0.348 ± 0.038 for the corresponding pregnant control women were statistically significantly different *(P *= 0.002). Using the lower 95% confidence interval of the mean expression level in PTL subjects (0.7) as a cutoff value for elevated TLR4 mRNA levels, 25/41 (60.9%) of PTL patients expressed elevated TLR4 mRNA as compared to 0/41 (0%) in control subjects. The TLR4 receptor levels in the granulocyte fraction of white blood cells from preterm labor and pregnant controls were similar. However, TLR4^+^/CD14^+^monocytes were 2.3 times more frequent (70% vs. 30%) and TLR4 also had a 2.6-fold higher density (750 vs. 280 molecules per cell) in preterm labor women compared with pregnant controls. There was no difference in the levels of TLR4 in patients at term.

**Conclusions:**

Patients with preterm labor exhibited elevated levels of CD14^+ ^maternal blood monocytes each bearing enhanced expression of TLR4, indicating that the peripheral circulatory system is activated in patients with preterm labor. Elevated leukocyte TLR4 levels may be a useful biomarker associated with preterm labor.

## Background

Preterm labor and delivery is a leading cause of prenatal morbidity and mortality worldwide, affecting approximately 12% of pregnant women in North America [1,2]. Furthermore, the first year of life medical cost for premature infants exceeds $8 billion annually [1,3]. Despite extensive research and aggressive medical management, the rate of preterm delivery has not decreased in the United States over the past 4 decades [4,5].

Subclinical urogenital infections have been implicated in up to 70% of preterm labor [6-8]. It is thought that a maternal inflammatory response leads to elevated levels of interleukins, tumor necrosis factor-α, and prostaglandins which contribute to the initiation of uterus contractility and preterm labor [9-12]. While the signaling pathways associated with the later stages of labor have been intensively investigated, the cascade of early signaling steps is not clear and requires further study.

As activation of the proinflammatory cascade may contribute to the onset of preterm labor, we sought to determine whether an innate immune response within the peripheral blood system was associated with the pathogenesis of preterm labor. Recent investigations provide evidence that initial host immune/inflammatory responses are controlled, in part, by a family of proteins known as toll-like receptors (TLRs). TLRs are expressed predominantly on monocytes within the peripheral blood system [13-15]. TLR4 plays a fundamental role in the early activation of innate immunity to exogenous and endogenous ligands including bacterial lipopolysaccharides, heat shock proteins, and components of the extracellular matrix released after tissue damage [16-21]. TLR4 signaling induces expression of a set of genes encoding proinflammatory cytokines (1L-1, 1L-6, and 1L-8), chemokines, and co-stimulatory molecules that can increase the level of prostaglandins, also recognized as effector molecules in preterm labor. TLR4 appears to be constitutively expressed in placental villous and intermediate trophoblasts and by macrophages (Hofbauer cells) located within the placental villi [22,23]. Increased expression of TLR4 in the villous Hofbauer cells has been observed in preterm placentas of patients with chorioamnionitis [22]. Whether the observed changes in TLR4 expression are limited to macrophages within the placental compartment or expressed more globally in the peripheral circulatory system is not clear.

To date, there are no comparative studies of TLR4 gene expression and flow cytometry/receptor density profiles of maternal peripheral blood monocytes during idiopathic preterm labor. Thus, the objective of the present study was to investigate whether the expression of TLR4 in maternal white blood cells in patients with idiopathic preterm labor is significantly elevated.

## Methods

### Subjects

The study was approved by the Human Research Committee at the University of Texas Medical Branch (UTMB) in Galveston, Texas and at Meharry Medical College, Nashville Tennessee. Written informed consent was obtained from all 159 recruited women. A case was defined as a pregnant woman who presented at the labor and delivery ward and was diagnosed by a physician as being in idiopathic preterm labor. The clinical criteria for preterm labor were those used by the American College of Obstetricians and included regular contractions, cervical dilation of 2 cm and/or cervical effacement. Exclusion criteria included maternal illness, anemia, uterine malformations, placental abruption, placenta previa, and steroid use. All women in preterm labor were clinically evaluated for symptoms of chorioamnionitis, bacterial vaginosis (BV) and urinary tract infection (UTI). BV was diagnosed based on clinical symptoms and by the evaluation of vaginal discharge including the presence of 20% clue cells. Women diagnosed with UTI, BV or chorioamnionitis were excluded. However, women with idiopathic PTL who developed clinical infection during their stay in L&D were included. Pregnant control patients were evaluated in a similar fashion during a prenatal clinical visit. In addition, exclusion criteria included patients who exhibited recurrent PTL, patients with a high risk of PTL, and patients who admitted to drug use. Controls were pregnant women not in PTL, who presented at the same hospital and were also subjected to the same exclusion criteria as the PTL cases. Both case and control populations were 18 years old or older. Neither cases nor controls were offered financial compensation for participation in the study. Cases and controls were limited to the following racial groups: white (non-Hispanic), black, and Hispanic. Both hospitals are state funded, have a primary population of the under-served, and provide free access to prenatal care.

Because the major goal of this investigation was to evaluate the TLR4 levels on peripheral blood, we only collected blood samples. The following clinical groups were evaluated: pregnant controls without preterm labor (n = 76) and women with PTL (weeks 24-36) (n = 63). The present analysis is restricted to the 41 controls and 41 cases who were evaluated for TLR4 gene expression after 23 weeks gestation; 25 controls and 12 PTL were tested for TLR4 receptor density analysis, and 10 controls and 10 PTL were tested for WBC count analysis.

To better understand the basic principles controlling TLR4 levels, we also evaluated 12 pregnant control subjects at term who were not in labor (weeks 39-40) and another 8 pregnant control patients at term who were in labor. These 20 patients presented at the same hospitals and were subject to the same exclusion criteria as the PTL and pregnant control groups.

The major goal of this study was to evaluate the association of TLR4 levels and PTL. In order to conduct the study, blood samples were collected from PTL cases and pregnant controls. Blood samples were collected from PTL cases prior to any treatments. Since PTL status and TLR4 levels were measured simultaneously, we do not imply causality and/or temporality and can only report PTL prevalence. This study is a cross-sectional analysis evaluating statistically significant differences of TLR4 expression levels between PTL cases and non-PTL pregnant controls.

### White blood cell and RNA isolation

Peripheral venous blood (5 mL) was drawn once into heparinized vacutainers from each case prior to treatment of PTL and from controls during a scheduled prenatal clinic visit. White blood cells were separated from erythrocytes by dextran sedimentation and pelleted by centrifugation; total RNA was isolated using Tri-Reagent (Sigma, St. Louis, Mo). Isolated RNA was quantified by optical density readings at 260 nm, and the purity was estimated by the ratio of 260/280 nm.

### Reverse transcriptase-polymerase chain reaction

The Dual Gene Quantitative (Maxim Biotech) and iQ SYBR Green Real Time PCR (Bio-Rad) methods were used to determine TLR4 mRNA levels. Isolated RNA was treated with RNAse-free DNase (Ambion, Austin, Tx) to ensure there was no contamination with genomic DNA. When the Dual Gene kit was used, 1 μg RNA was reverse transcribed using Moloney-Murine Leukemia Virus reverse transcriptase (RT) and random decamer primers using the manufacturer's protocol (RETROscript Kit, Ambion). Dual quantitative polymerase chain reaction (PCR) amplification was performed using 5 μL of cDNA (~0.25 μg), 1.5 U of Taq polymerase (Life Technologies, Carlsbad, Calif.), and PCR thermal cycler (Thermo Hybrid, Franklin, Mass.). The TLR4 primer sequences were TLR4 forward-5'-GGA AGT TGA ACG AAT GGA ATG TG-3' and TLR4 reverse 5'-TCT GAG TCG TCT CCA GAA GIT GTG-3'. The primer sequences for the 18S rRNA gene were not disclosed by the company. The optimized amplification protocol (96°C for 1 minute, 36 cycles of 94°C for 1 minute, 60°C for 90 seconds followed by a final extension at 72°C for 10 minutes) allowed good linear range amplification of TLR4. The observed differences between replicates from the same women were less than 10% and on average varied from 2% to 5%. Of several housekeeping genes evaluated, we selected the 18S rRNA gene since its expression remains constant during pregnancy. As the number of 18S copies greatly exceeds that of TLR4, the amount of 18S was determined using a 1:500 dilution of the rtRNA with linear range amplification at cycle 14.

Real time RT-PCR was performed using the iScript cDNA synthesis and iQ SYBR green reaction mixes and analyzed using the Bio-Rad iCycler apparatus. For this, 600 ng of RNA was reverse transcribed using the iScript cDNA synthesis kit in a total volume of 25 ul. For TLR4, 2 ul of cDNA reaction mix (48 ng) was subjected to PCR analysis in a total volume of 25 ul: 2 ul of a 1:500 diluted sample was used to determine the number of 18S rRNA copies. TLR4-specific oligos hybridized within exons 2 and 3 were mRNA-specific, generated a 236 bp fragment, and exhibited a 94.5% PCR efficiency. The sequences of the TLR4 forward and reverse oligo's were 5'-AAA ATC CCC GAC AAC CTC CC and 5'-AGT CCA GAA AAG GCT CCC AGG, respectively. The TLR4 mRNA pool sizes were normalized using 18S rRNA as the internal standard using oligonucleotides 18Sf 5'-CGG ACA GGA TTG ACA GAT TGA TAG C and 18Sr 5'-TGC CAG AGT CTC GTT CGT TAT CG. The 18S amplicon was an 118 bp fragment and exhibited an 81% PCR efficiency. During the course of our analysis, the specificity of the amplification was determined by melt curve analysis and by visualization using agarose gel fractionation.

### Quantitation of RT-PCR products

For the Dual Gene kit, the intensities of the PCR products were digitally captured and quantitated using an AlphaImager HP image-scanning system (Alpha Innotech Corporation, San Fernando, Calif.). The normalized expression of the TLR4 gene was calculated as follows: X/Y = standardized TLR4 gene expression, where X = TLR4 gene expression level, and Y = 18S ribosomal gene (18S) expression level.

For the iCycler real time analysis, the changes in TLR4 expression were determined using the absolute standard curve method normalized using 18S rRNA. TLR4 and 18S copy numbers were determined by extrapolation from a standard curve generated using linearized plasmid DNA (pCR2.1) that contained the TLR4 and 18S rRNA amplicon, respectively. The correlation coefficients of the standard curves were >0.99 and encompassed the entire range of experimental copy numbers. The Ct threshold values were in the 200 - 300 range.

### Flow cytometry & quantitation of TLR4 receptor molecules expression in patients WBC's

White blood cells isolated from pregnant women were resuspended at 5 × 10^6 ^cells/mL in the staining buffer (phosphate buffered saline without Ca2+ and Mg2+, 1% BSA, 0.05% sodium azide). 50 μL aliquots were stained with anti-TLR4 (IgG2a) monoclonal antibody conjugated with phycoerithrin (PE, 80 μg/mL; Santa Cruz Biotechnology, Santa Cruz, Calif.) and anti-CDI4 IgG2a conjugated with fluorescein (BD PharMingen, San Diego, Calif.), and incubated on ice for 30 minutes in the dark. Isotype-matched irrelevant antibody controls were used to detect nonspecific staining. Cells were washed three times, pelleted by centrifugation (1,000 × g for 2 minutes at 4°C), and resuspended in staining buffer. Staining was performed in the presence of 100 μg/mL of human IgG to block nonspecific binding to Fc_ receptors. Cells were analyzed by flow cytometry on a Becton Dickinson FACScan microfluorometer (Becton Dickinson & Co., Franklin Lakes, NJ). The percentage of TLR4+ granulocytes and monocytes was determined per 15,000 events analyzed using forward (FSC) and side (SSC) scattering or FSC, SSC and CD14 staining respectively. Data were analyzed with CellQuest

software (BD Bioscience, San Jose, Calif.). The quantitation of the number of monocyte TLR4 receptors was conducted using anti-TLR4 mAb-PE conjugates and QuantiBRITE PE beads (Becton Dickinson & Co) as recommended by the manufacturer.

### Statistical analysis

In order to estimate the required sample size, *a priori *power analysis was conducted with G*Power software version 3 [24], using a two-tailed t-test with an alpha error probability of 0.05 and an effect size of 0.5. The results were that 26 patients (13 cases and 13 controls) would be required to achieve 80% power and 42 patients (21 cases and 21 controls) would be required in order to achieve 95% power.

Demographic variables are presented as medians and interquartile ranges (IQR) or as numbers and percents. Differences in demographic characteristics of PTL and pregnant controls were assessed using Mann-Whitney tests and Kruskal-Wallis tests. Multiple linear regression was used to compare the mean TLR4 gene expression of PTL and pregnant controls while adjusting for confounding. According to a normal probability plot TLR4 gene expression was not normally distributed, therefore we performed Box-Cox regression to determine the most appropriate transformation. A reciprocal transformation of TLR4 gene expression was used for the regression analysis, and the estimated means were then retransformed for presentation. Statistical analysis was conducted with STATA Software, (Stata Corp, College Station, Texas). A *P*-value of less than 0.05 was considered significant.

## Results and Discussion

### **Baseline characteristics of the patient population**

Demographic and clinical data for patients with preterm labor (PTL) (n = 41) and controls (n = 41) used to evaluate peripheral blood system TLR4 mRNA levels are summarized in Table 1. Demographic and clinical data for the subsets of controls who had term labor (n = 8) or did not have term labor (n = 12) used to determine TLR4 expression during the course of spontaneous term labor are also summarized in Table 1. Although women included in the preterm labor and pregnant control groups were similar in terms of maternal age, race, parity, marital status, educational level, and smoking status we adjusted for maternal race and smoking in the regression analysis.

**Table 1:** Demographic characteristics of study population by preterm labor

Characteristics	Preterm labor	Pregnant	P-value
	(n = 41)	(n = 41)	
	No. (%)	No. (%)	
Age (median, IQR)	23 (7)	24 (8)	0.32
Race			
White non-Hispanic	13 (31.7)	11 (29.7)	0.27
Black	11 (26.8)	4 (10.8)	
Hispanic	22 (41.5)	22 (59.5)	
Missing	4	0	
Parity (median, IQR)			
Married			
Years of education (median, IQR)			
Cigarette smoker	9 (22.0)	7 (17.1)	0.58

**Characteristics**	**Pregnant term labor**	**Pregnant not term labor**	**P-value**
	**(n = 8)**	**(n = 12)**	
	**No. (%)**	**No. (%)**	

Age (median, IQR)	25 (3)	24 (10)	0.99
Race			
White non-Hispanic	1 (31.7)	1 (14.3)	0.89
Black	0 (0.0)	2 (28.6)	
Hispanic	5 (41.5)	4 (57.1)	
Missing	2	5	
Parity (median, IQR)			
Married			
Years of education (median, IQR)			
Cigarette smoker	0 (0.0)	0 (0.0)	---

### Assessment of TLR4 mRNA levels in peripheral blood of PTL patients

**Figure 1:**
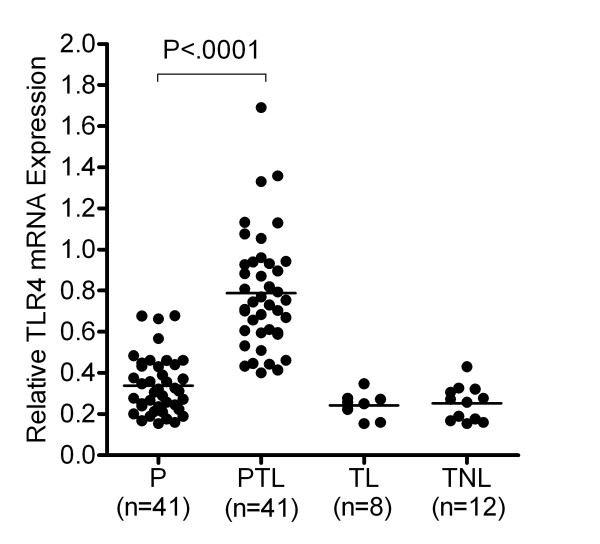
**Expression of TLR4 mRNA in maternal peripheral blood leukocytes in pregnant controls and in women with preterm or term labor**. TLR4 mRNA levels was determined relative to 18S rRNA using a quantitative dual gene approach and expressed as the integrated density value of the amplicon. The expression levels of pregnant (P) and woman with spontaneous preterm labor (PTL) were significantly different *(P *= 0.002). The expression levels of the term non-labor (TNL), and term labor (TL), were not significantly different (p > 0.05).

The median gestational ages when the samples were collected, 31.2 (IQR 10) for the PTL group and 31.4 (IQR 6.2) for the control subjects, were not statistically significantly different (*P *= 0.62); however, additional adjustment was made for this variable in the regression analysis. Adjusted mean TLR4 mRNA levels (Figure 1) of 0.788 ± 0.037 (standard error) for preterm labor and 0.348 ± 0.038 for the corresponding pregnant control women were statistically significantly different *(P *= 0.002). Using the lower 95% confidence interval of the mean expression level in PTL subjects (0.7) as a cutoff value for elevated TLR4 mRNA levels, 25/41 (60.9%) of PTL patients expressed elevated TLR4 mRNA as compared to 0/41 (0%) in control subjects.

We also determined if TLR4 expression within the peripheral blood system was elevated during the course of spontaneous term labor (gestational age 39-40 weeks). The TLR4 mRNA levels (0.24 ± 0.02) in term labor women (n = 8) was not statistically different from the levels (0.25 ± 0.02) observed in term pregnant women not in labor (n = 12) and both populations exhibited TLR4 levels similar to that observed in the control subjects (Figure 1). We conclude that TLR4 expression in peripheral white cells is not elevated during spontaneous term labor.

We performed parallel experiments using quantitative real time RT-PCR technology. For this we empirically engineered and utilized TLR4 primers that were mRNA-specific. Experiments were repeated 3 times and the absolute quantitative method was used to determine copy numbers of TLR4 and 18S rRNA per ug input reverse-transcribed total RNA. The PCR efficiencies of the standards were similar in control reactions performed in the presence of non RT-RNA and no corrections for PCR efficiencies were required in our determination of the number of TLR4 mRNA copies within each sample normalized to 18S rRNA. The only assumption we made is that the reverse transcription efficiencies of the samples were similar. All amplification reactions were also visually checked to verify specificity. The results using quantitative real time RT-PCR were consistent with the data obtained using quantitative dual gene PCR giving a 2.2-fold elevated expression level of TLR4 in preterm labor patients.

### TLR4 levels in PTL patients diagnosed with infection

Among 41 idiopathic preterm labor women, during hospitalization, 10 women developed either chorioamnionitis or urinary tract infection and 1 woman developed both. When the patients with known infections were removed from the preterm labor group, the adjusted mean level of TLR4 mRNA was increased from 0.782 ± 0.036 to 0.789 ± 0.037 (n = 31), *(P *< 0.0001). Therefore, an increase in TLR4 mRNA levels was observed even in those PTL patients without clinical signs of an infection. None of the patients in the control group were diagnosed with clinical infection of the urogenital tract.

### Localization and quantification of TLR4 receptors on peripheral blood leukocyte populations

To investigate whether the observed enhanced expression level of TLR4 mRNA in PTL subjects was due to elevated WBC counts, 10 random (gestational age 24-36 weeks) pregnant controls and 10 random preterm labor women (gestational age 24-36 weeks) were analyzed. The mean white blood cell count of 10.47 × 10^6^/ml for the pregnant controls and 9.38 × 10^6^/ml for the preterm labor group indicated that there was no significant difference between these groups (*P *> 0.05). This suggests that the observed difference in the expression level of TLR4 between PTL and control patients cannot be explained by an overt increase in white blood cell (WBC) counts.

**Figure 2:**
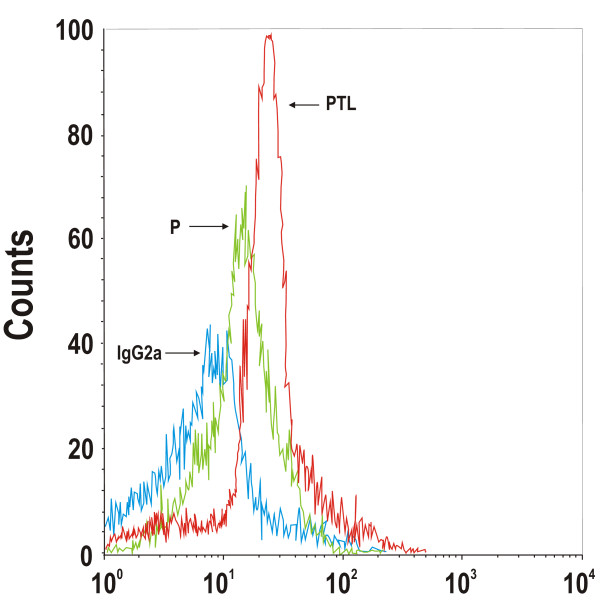
**TLR4 surface expression on peripheral blood monocytes in pregnant control (P), and preterm labor (PTL) women**. This illustrative histogram depicts TLR4 staining between pregnant and premature labor subjects and the nonspecific staining observed using the isotype control (IgG2a) antibody. The events are indicated on the y-axis and the anti-TLR4-phycoerithrin fluorescence intensity of gated CD14^+ ^monocytes (as determined by anti-CD14-fluorescein fluorescence and FSC/SSC) was plotted along the x-axis. The mean data ± SEM for each group, % of TLR4 positive monocytes and number of molecules per cell are included in Table 2.

We next determined if the observed changes in mRNA pool sizes reflected changes in the expression profile of the TLR4 molecules on the surface of peripheral blood leukocytes. In particular, TLR4 receptors have been shown to be expressed on both subpopulations of neutrophils and basophils (granulocytes) and on non-granulocyte circulating monocytes [13].Using a total of 25 random pregnant controls (gestational age 24-36 weeks) and 12 random preterm labor subjects (gestational age 24-36 weeks), we used flow cytometry gating and anti-TLR4 staining to analyze TLR4 protein expression on granulocytes. We observed that 2.7% of granulocytes in preterm labor were TLR4-positive (geometric mean fluorescence [GMF] of 7.65 ± 0.37) (forward [FSC] and side [SSC] scattering plots and fluorescence data not shown). In pregnant controls, 2.3% of granulocytes were positive for TLR4 (GMF of 6.46 ± 0.24). The observed difference was not statistically significant (*P *> 0.05). By contrast, analysis of maternal monocytes, identified by FSC/SSC gating and CD14 staining showed that the preterm labor group contained 70% TLR4-positive monocytes (GMF of 14.8 ± 3.92), whereas the pregnant control group contained 30% TLR4-positive cells (GMF of 7.9 ± 0.33) (Table 2). A representative gated monocyte TLR4 staining profile of pregnant and preterm labor cohorts is shown in Figure 2. In addition, quantitative fluorescence flow cytometry indicated that the preterm labor monocytes expressed TLR4 in the range of 750 ± 75 molecules per cell, whereas, the pregnant control group expressed roughly 280 ± 20 receptors per cell (Table 2). These results suggest that on average, the total number of TLR4 molecules in the maternal peripheral CD14^+ ^monocyte pool of PTL subjects was more than 5 times higher than in control subjects, when considering that both the absolute number of monocytes as well as the surface expression density of TLR4 per monocyte was increased in the PTL subjects.

**Table 2:** TLR4 receptor density in peripheral blood monocytes of pregnant women and women with spontaneous preterm labor.

	% of TLR4 Positive Monocytes	Receptors per Cell	Monocytes Mean Fluorescence
Pregnant Group (n = 10)	30	280 ± 20	7.9 ± 0.332
PTL Group (n = 10)	70	750 ± 75*	14.8 ± 3.921*

*p < 0.05 vs. pregnant group, One-way ANOVA with Newman-Kuels test

## Conclusions

Localized infection-associated intrauterine recruitment/activation of neutrophils and monocytes has been implicated in the pathogenesis of preterm labor in humans (see [25] and references therein). While the coordinated molecular pathways that regulate the recruitment and activation of granulocytes and non-granulocytes are complex, monocytes bearing the proinflammatory signal transducer TLR4 receptor appear to have a critical role in the orchestration of the leukocyte response to gram-negative microbial molecular patterns [13,26]. To date, monocyte activation in the peripheral blood system of preterm labor patients has not been carefully examined.

To our knowledge, this is the first report that characterizes TLR4 expression in peripheral blood monocytes and granulocytes during pregnancy with or without preterm labor. We observed a significant (over 2-fold) increase in TLR4 mRNA expression in women undergoing spontaneous preterm labor compared with pregnant controls. At the cellular and protein levels, roughly 3% of granulocyte cells expressed TLR4 on their surface, and the expression level was similar in control and PTL patients. By contrast, on average, the percentage of monocytes that expressed TLR4 receptor was approximately double that seen in the PTL patients (70% of the monocytes were TLR4+) and these monocytes expressed roughly 2.5-fold more TLR4 receptors per cell than those within the pregnant control subjects. The elevated level of TLR4 was not associated with leukocytosis, as the overall number of leukocytes did not change significantly, when comparing PTL and controls.

An intriguing aspect of our study is that more than 60% of subjects with idiopathic PTL exhibited a significant increase in TLR4 expression in peripheral WBC's, yet did not display clinical indications of infection. One simple explanation of our results is that the PTL subjects that exhibit an elevation of TLR4 in their accessible peripheral circulation may suffer from either a subclinical infection and/or a non-infectious inflammatory process.

Our data suggest that the process of labor, per se, does not lead to an elevation of TLR4 mRNA levels in peripheral WBC's (Figure 1). This finding is in agreement with the recent study of Haddad et. al., who did not detect changes in TLR4 expression within peripheral blood RNA generated from patients at term with no labor and at term with labor. [27] Therefore, elevated TLR4 expression within peripheral WBCs may serve as a useful marker for PTL.

Consistent with our data reported here (60.9% of PTL patients expressed elevated TLR4 as compared to 0/41 (0%) in control subjects), we have recently demonstrated that CD55 (a key regulator of the complement cascade and an apparent WBC biomarker for inflammation [28]) was expressed at elevated levels in the peripheral WBC's of 71% of tested PTL patients as compared to 6% of pregnant control patients [29]. Taken together, these studies strongly support the notion that the peripheral circulatory system in the majority of PTL subjects is immunologically "activated" [30]. It should be pointed out that while sensitivity and specificity of our current TLR4 (data not shown) and published CD55 studies were very promising this study has some limitations including the sample size of subgroups and difference in proportion of black patients in the PTL and control groups. Nevertheless, this study paves the way for a prospective cohort study. A much larger data set will be required to evaluate with confidence the diagnostic potential of TLR4 and CD55 as predictors of preterm delivery. Use of elevated peripheral blood levels of either TLR4 or CD55 or both as a diagnostic tool for preterm labor will require larger sample sizes and a more thorough understanding of their temporal expression patterns.

In summary, while there is ample evidence that intrauterine inflammatory responses contribute to the pathophysiology of preterm labor, it is generally accepted that these responses are confined to the reproductive unit. We have uncovered evidence that the maternal peripheral blood system of patients with preterm labor is in a more immunologically "active" state than that observed in pregnant woman. While the significance of the observed enhanced TLR4 expression within circulating monocytes in women with preterm labor requires further analysis to delineate what effects they might have on the mechanism of PTL, it is intriguing to hypothesize that trafficking of the "inflammatory" monocytes through the peripheral circulatory system may contribute to the pathophysiology of preterm labor. We anticipate that these finding will serve as a foundation to develop new strategies for early detection and prevention of this extremely burdensome socioeconomic disease.

## Abbreviations

TLR: toll-like receptor; BV: bacterial vaginosis; PTL: preterm labor; FSC: forward light scattering; SSC: side light scattering; IDV: integrated density value; UTI: urinary tract infection; WBC: white blood cells; GMF: geometric mean fluorescence.

## Competing interests

The authors declare that they have no competing interests.

## Authors' contributions

SN, BN, MI have full access to all of the data in the study and take responsibility for the integrity of the data and the accuracy of the data analysis. All authors read and approved the final manuscript.

SN, BN, EP developed study concept and design. EP conducted acquisition of data.

EP, BN, MI, SP conducted analysis and interpretation of data. EP, BN, MI, SP, SN wrote the first draft of the manuscript. BN, MI, SN provided critical revision of the manuscript for important intellectual content. MS, NS, EP performed statistical analysis. BN, SN obtained funding. BN, MI, SN provided administrative, technical, or material support. BN, SN provided study supervision.

## Author Information

Edyta Pawelczyk (The National Institutes of Health, Bethesda,

Maryland); Bogdan J. Nowicki (The Departments of Obstetrics & Gynecology and the Department of Microbiology and Immunology, Meharry Medical College, Nashville, TN), Michael G. Izban, and Stella Nowicki (the Department of Microbiology and Immunology, Meharry Medical College, Nashville, TN); Maureen Sanderson (The Department of Obstetrics & Gynecology), Siddharth Pratap (the Department of Microbiology and Immunology, Meharry Medical College, Nashville, TN), Nupur A. Sashti (Communicable and Environmental Disease Services, Tennessee Department of Health, Nashville, TN.).

## Pre-publication history

The pre-publication history for this paper can be accessed here:

http://www.biomedcentral.com/1471-2393/10/66/prepub
